# Successful Endoscopic Vacuum Therapy for a Postoperative Esophagogastric Fistula Following Sleeve Gastrectomy: A Case Report

**DOI:** 10.7759/cureus.88944

**Published:** 2025-07-28

**Authors:** Antonio de Jesús González Luna, Marco Antonio Castellanos López, Luis Antonio Velázquez Lozano, Nancy Cristina Vela Moya, Cristina Vanessa Cuevas Calla

**Affiliations:** 1 Department of General Surgery, Regional Hospital “Dr. Valentin Gomez Farias”, Institute of Security and Social Services for the State Workers (ISSSTE), Zapopan, MEX; 2 Department of General Surgery, National Polytechnic Institute (IPN), Mexico City, MEX; 3 Department of General Surgery, General Hospital Zone No. 3, Mexican Institute of Social Security (IMSS), Aguascalientes, MEX; 4 Department of General Surgery, University of Guadalajara (UDG), Zapopan, MEX

**Keywords:** endoscopic negative pressure therapy, gastroesophageal fistula, negative-pressure wound therapy, postoperative complications, sleeve gastrectomy, upper gastrointestinal endoscopy

## Abstract

Esophagogastric fistulas are a rare but serious complication after sleeve gastrectomy. Their management remains a clinical challenge, especially when conventional endoscopic approaches, such as stent placement, fail. We report the case of a 49-year-old female with a history of hypothyroidism, major depressive disorder, body mass index greater than 50 kg/m², significant smoking history, and obstructive sleep apnea, who developed acute abdominal pain and bleeding after laparoscopic sleeve gastrectomy. She was referred to our institution in hypovolemic shock and underwent exploratory laparotomy with abdominal packing. An upper endoscopy revealed a 5-10 mm esophagogastric leak initially managed with a covered self-expanding stent, which subsequently migrated. The persistent fistula was confirmed by contrast study, and endoscopic negative pressure therapy (endoscopic vacuum-assisted closure (EndoVAC)) was initiated using a polyurethane sponge with scheduled replacements. After six sessions, complete fistula closure was achieved, confirmed by endoscopic and radiographic evaluation. This case highlights the successful use of EndoVAC therapy as a minimally invasive and effective option for managing refractory post-bariatric esophagogastric fistulas. Early consideration of this technique may improve outcomes in similar scenarios.

## Introduction

Leaks and fistulas following bariatric surgery, particularly after sleeve gastrectomy, are uncommon but potentially serious complications, with a reported incidence ranging from 0.7% to 5.3% [1]. These complications prolong hospital stays, increase morbidity, and may necessitate reoperation, significantly affecting patient recovery and quality of life.

Traditional treatment modalities include self-expanding metal esophagogastric stents, internal drainage techniques, or endoscopic clips. However, these approaches are often limited by device migration, partial failure, or defect recurrence [2]. In recent years, endoscopic vacuum therapy (EVT) (or endoscopic vacuum-assisted closure (EndoVAC)) has emerged as a reliable and less invasive alternative. A recent meta-analysis reported a clinical success rate of 87.2%, with a moderate adverse event rate of 6% and a system displacement rate of 12.5% [3]. Similarly, studies focusing on leaks related to bariatric surgery have shown an efficacy of approximately 90%, with an average of six to seven sessions required for complete defect closure [3]. Endoscopic treatment of leaks has been associated with higher closure rates compared to fistulas [4].

The EndoVAC procedure involves endoscopic placement of a polyurethane sponge connected to a nasogastric drainage tube directly into the esophagogastric fistula. The system is adjusted to deliver intermittent suction at approximately 100 mmHg using a portable vacuum unit, consistent with recent clinical practice [5]. Sponge exchanges are typically performed every three to five days under endoscopic guidance until fistula closure is confirmed.

Nevertheless, clinical experience with EndoVAC for fistulas specifically located at the esophagogastric junction following sleeve gastrectomy remains limited. This case report aims to expand the medical literature by presenting a successful example in which six EndoVAC sessions achieved complete resolution of a postoperative esophagogastric fistula, without complications or the need for additional surgical intervention, with outcomes confirmed by endoscopic and contrast imaging evaluations.

## Case presentation

We present the case of a 49-year-old female patient with a medical history of hypothyroidism and major depressive disorder who underwent laparoscopic sleeve gastrectomy at a private institution. The patient had a body mass index (BMI) greater than 50 kg/m², a history of heavy smoking, and was diagnosed with obstructive sleep apnea, all recognized risk factors for postoperative complications following bariatric surgery.

In the immediate postoperative period, she developed acute abdominal pain and hemorrhage through a Blake drain. Three days later, she was referred to our center in a state of hypovolemic shock. Initial fluid resuscitation was performed, followed by urgent transfer to the operating room.

During exploratory laparotomy, no clear source of active bleeding was identified, and abdominal packing was performed. A second surgical intervention was carried out 48 hours later for pack removal, with no evidence of further bleeding. The patient remained hospitalized with stable clinical progress.

**Figure 1:**
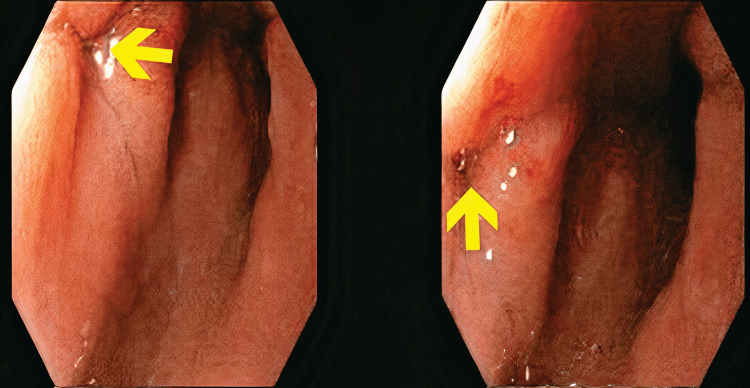
Initial Endoscopic Evaluation of the Esophagogastric Leak. Paired endoscopic images revealing a mucosal defect located approximately 1 cm distal to the esophagogastric junction. The fistulous opening measures between 5 and 10 mm in diameter and displays edematous margins with mucous exudate. These findings are consistent with an acute postoperative leak following laparoscopic sleeve gastrectomy.

**Figure 2:**
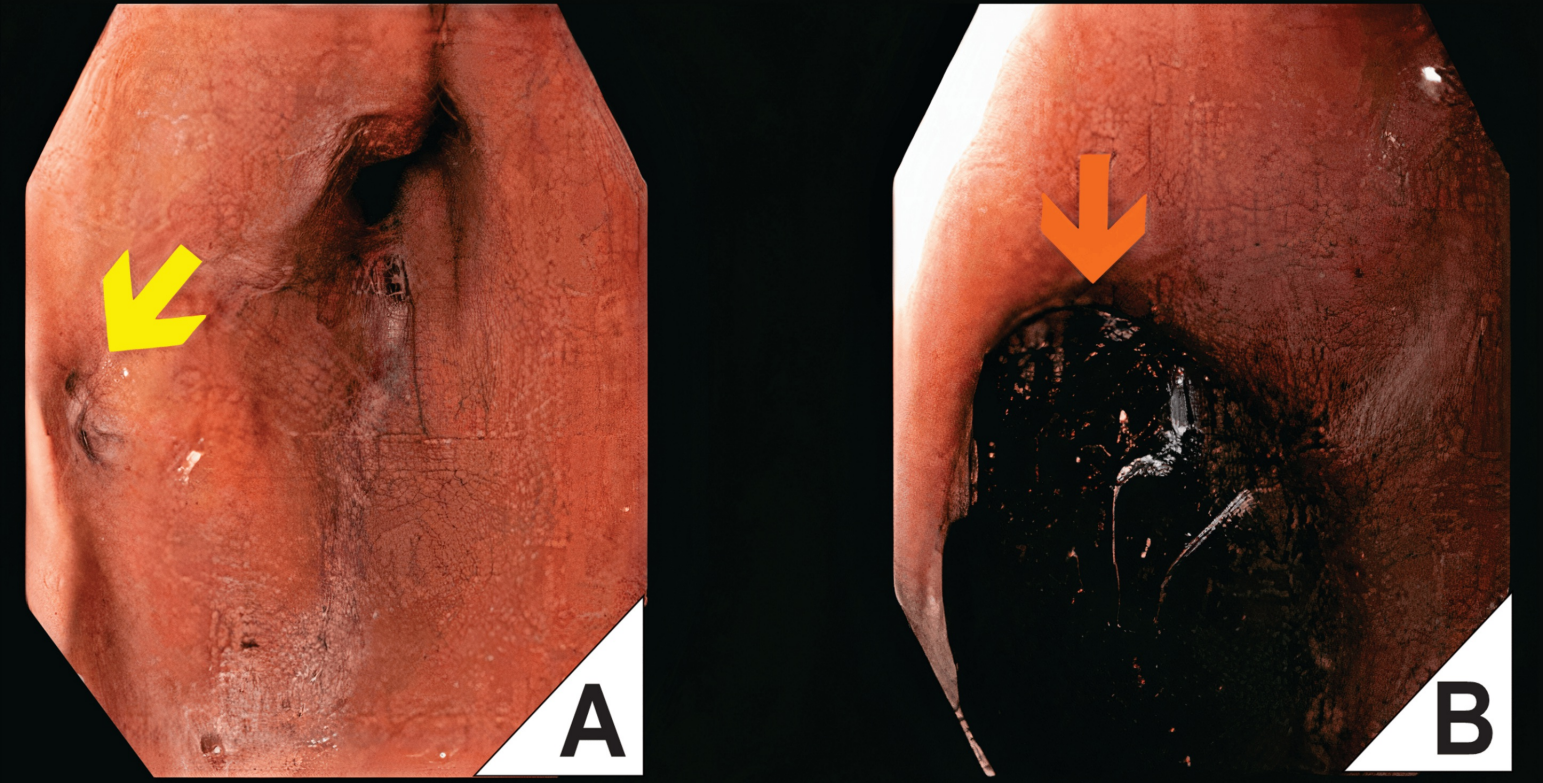
Endoscopic Placement of a Covered Self-Expandable Metal Stent (SEMS) for Esophagogastric Leak Management. (A) Endoscopic view showing a persistent fistulous opening at the esophagogastric junction, with surrounding mucosal edema, inflammation, and foamy exudate consistent with ongoing leakage. (B) Under direct endoscopic visualization, a covered self-expandable metal stent (orange arrow) is placed as an initial therapeutic attempt to seal the defect.

**Figure 3:**
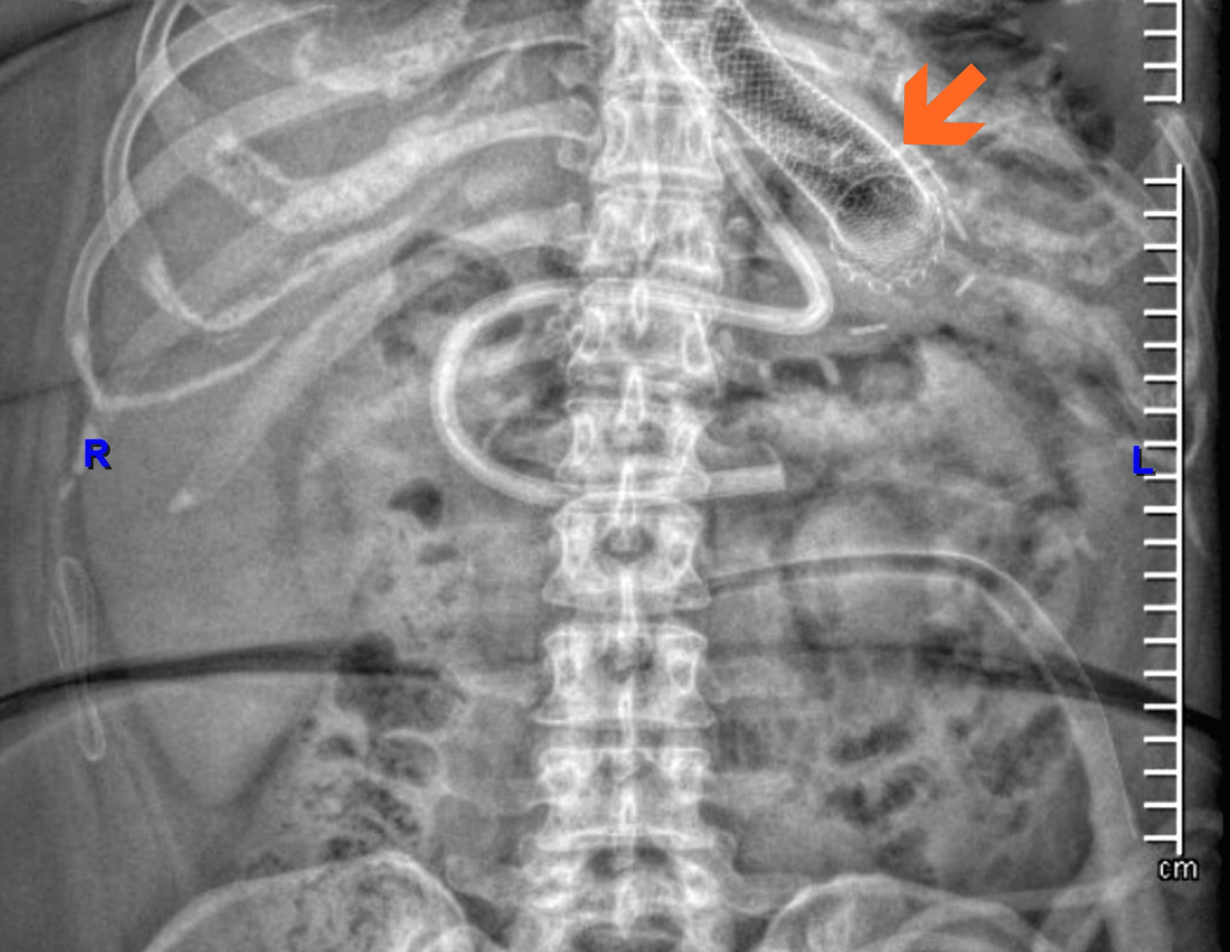
Plain Abdominal Radiograph After Placement of Covered Self-Expandable Esophagogastric Stent. Anteroposterior plain abdominal radiograph showing a covered self-expandable metallic stent in an appropriate intraluminal position at the esophagogastric junction. No signs of migration or pneumoperitoneum are observed. Intra-abdominal tubes and drains are also visible, consistent with ongoing postoperative management.

**Figure 4:**
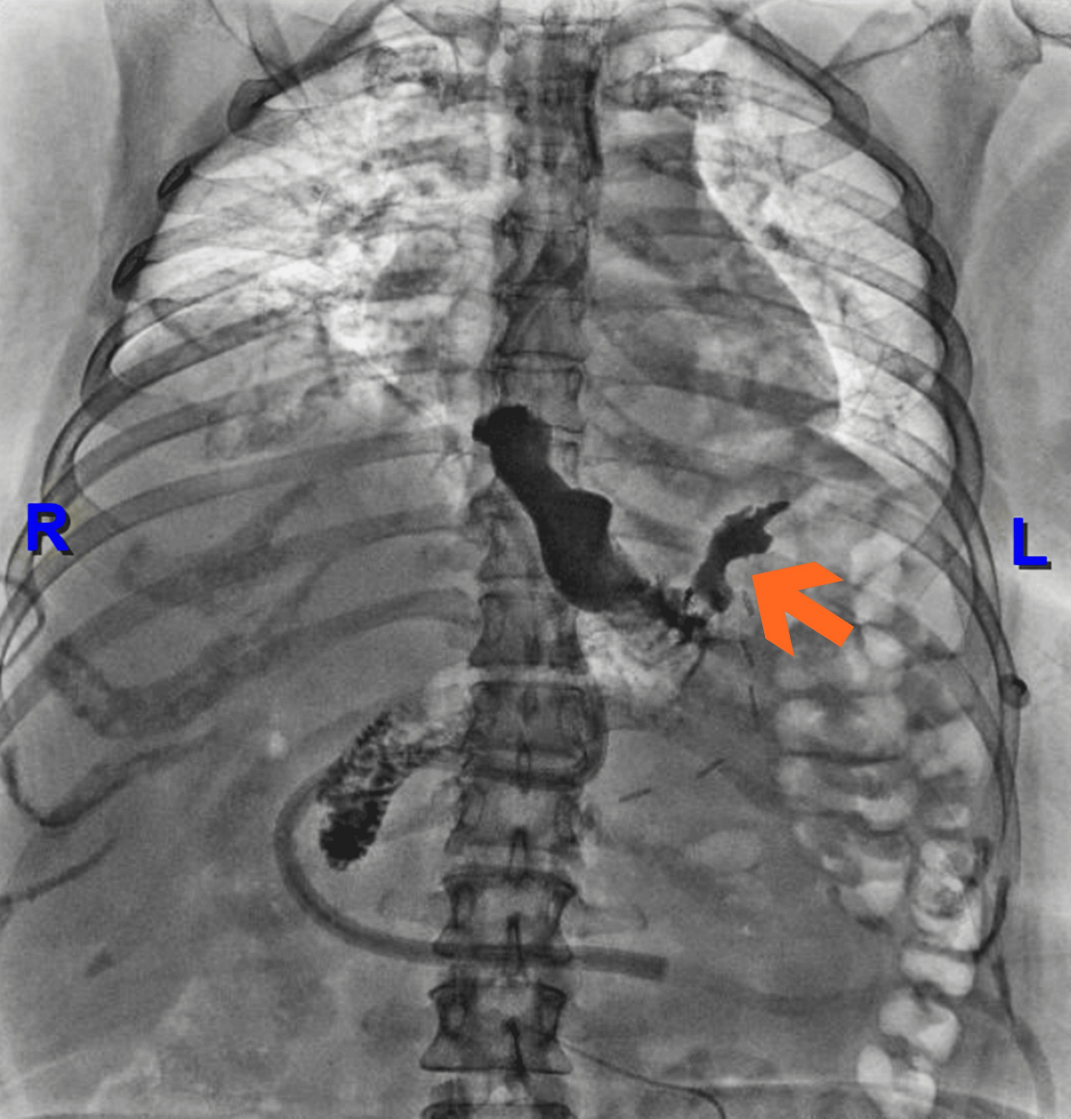
Upper Gastrointestinal Series With Contrast Medium. Fluoroscopic image demonstrating oral contrast administration with passage into the stomach and contrast extravasation at the esophagogastric junction, consistent with an esophagogastric fistula. A fistulous tract extending into the adjacent extraluminal space is observed.

An upper gastrointestinal endoscopy revealed an acute esophagogastric leak measuring approximately 5 to 10 mm in diameter, located 1 cm distal to the esophagogastric junction, within the proximal portion of the gastric sleeve. (Figure 1). A fully covered self-expanding metal stent (SEMS) was placed with therapeutic intent, followed by a plain abdominal X-ray to confirm positioning (Figures 2-3). However, three days later, the patient was readmitted due to severe abdominal pain. Repeat endoscopy showed stent migration and persistent leakage. The stent was removed, and an esophagogastroduodenal contrast study confirmed the persistence of a fistulous tract (Figure 4).

**Figure 5:**
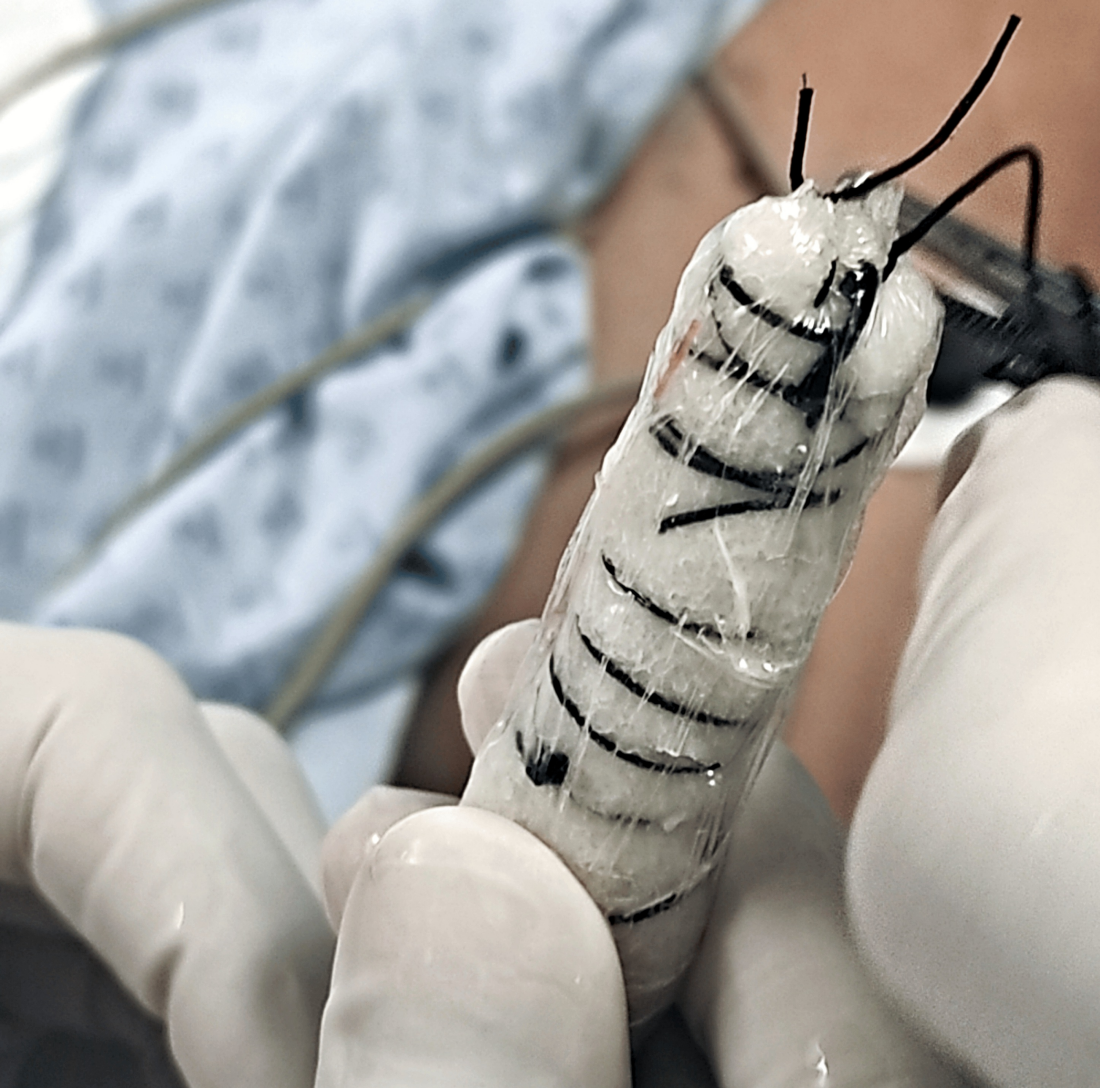
EndoVAC Device Prepared for Endoscopic Insertion. Clinical image displaying a white polyurethane sponge wrapped around a nasogastric drainage tube and secured with non-absorbable suture. This system, known as EndoVAC, is connected to a negative pressure unit to promote closure of esophagogastric fistulas. It is utilized in the management of postoperative leaks with endoscopic placement and exchange every three to five days. EndoVAC, endoscopic vacuum-assisted closure

**Figure 6:**
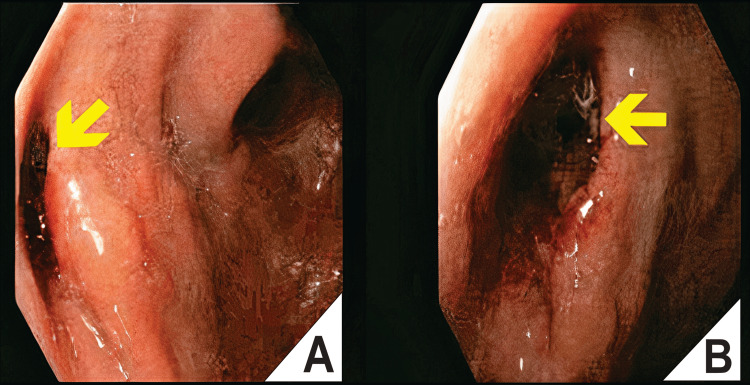
Endoscopic Progression of Esophagogastric Fistula Closure Using EndoVAC Therapy. Paired endoscopic images (A and B) showing the esophagogastric fistula captured from two distinct perspectives: (A) lateral view, revealing the fistulous orifice at the esophagogastric junction (yellow arrow), with surrounding mucosal edema and granulation tissue; (B) Frontal view of the same lesion, highlighting the fistulous opening (yellow arrow) and its relationship to adjacent mucosa. These images were obtained during ongoing EndoVAC therapy, documenting the fistula's morphology and position. EndoVAC, endoscopic vacuum-assisted closure

Given the persistent defect and clinical progression, a diagnosis of postoperative esophagogastric fistula was established. EndoVAC was initiated using a polyurethane sponge connected to a continuous suction system (Figure 5). The device was replaced endoscopically every three to five days. Significant clinical improvement was observed after the third session, with progressive closure of the fistulous tract (Figure 6). During the entire EndoVAC treatment, the patient remained hospitalized in a surgical unit under close monitoring. Initially, the patient was kept nil per os (NPO) and received total parenteral nutrition (TPN). As clinical improvement was noted, a nasojejunal feeding tube was placed during one of the sponge replacements to initiate enteral nutrition. This approach allowed for better caloric intake and the delivery of essential nutrients to support tissue healing, thereby enhancing the effect of the negative pressure therapy. Once fistula closure was confirmed by contrast imaging, oral intake was gradually reintroduced without complications.

During the acute phase, prior to the initiation of EndoVAC therapy, laboratory studies revealed leukocytosis of 18,200 × 10³/μL (reference range: 4.50-10.50), neutrophilia of 11.90 × 10³/μL (2.50-7.00), and stable hemoglobin levels of 13 g/dL (12.6-16.6). Renal function and electrolyte levels remained within normal limits (Table 1).

**Table 1:** Laboratory Results With Reference Ranges.

Parameter	Patient Value	Reference Range
Red Blood Cells (RBC)	5.04 ×10⁶/μL	4.20-5.40
Hemoglobin (Hb)	13.0 g/dL	12.6-16.6
Hematocrit (Hct)	40.2%	36.6-47.3
Platelets (PLT)	612 ×10³/μL	150.0-420.0
White Blood Cells (WBC)	18.20 ×10³/μL	4.50-10.50
Absolute Neutrophil Count (ANC)	11.90 ×10³/μL	2.50-7.0
Glucose	107 mg/dL	74.0-109.0
Blood Urea Nitrogen (BUN)	14.8 mg/dL	6.0-20.0
Creatinine	0.59 mg/dL	0.50-0.90
Total Bilirubin	0.39 mg/dL	0.00-1.20
Direct Bilirubin	0.2 dL	0.00-0.20
Indirect Bilirubin	0.19 mg/dL	0.30-0.80
Alanine Aminotransferase (ALT)	11 U/L	10.0-35.0
Aspartate Aminotransferase (AST)	27 U/L	10.0-36.0
Lactate Dehydrogenase (LDH)	182 U/L	135.0-214.0
Calcium	8.8 mg/dL	8.4-10.2
Phosphorus	3.6 mg/dL	2.5-4.5
Chloride	104 mEq/L	98-107
Potassium	4.5 mEq/L	3.5-5.1
Sodium	145 mEq/L	136-145
Magnesium	2 mg/dL	1.6-2.3
Lactate	0.70 mmol/L	0.30-0.70

In the following days, concurrent with the patient’s clinical improvement, there was a progressive normalization of inflammatory markers. Leukocyte and neutrophil counts returned to reference values, indicating a favorable systemic response to the implemented treatment.

**Figure 7:**
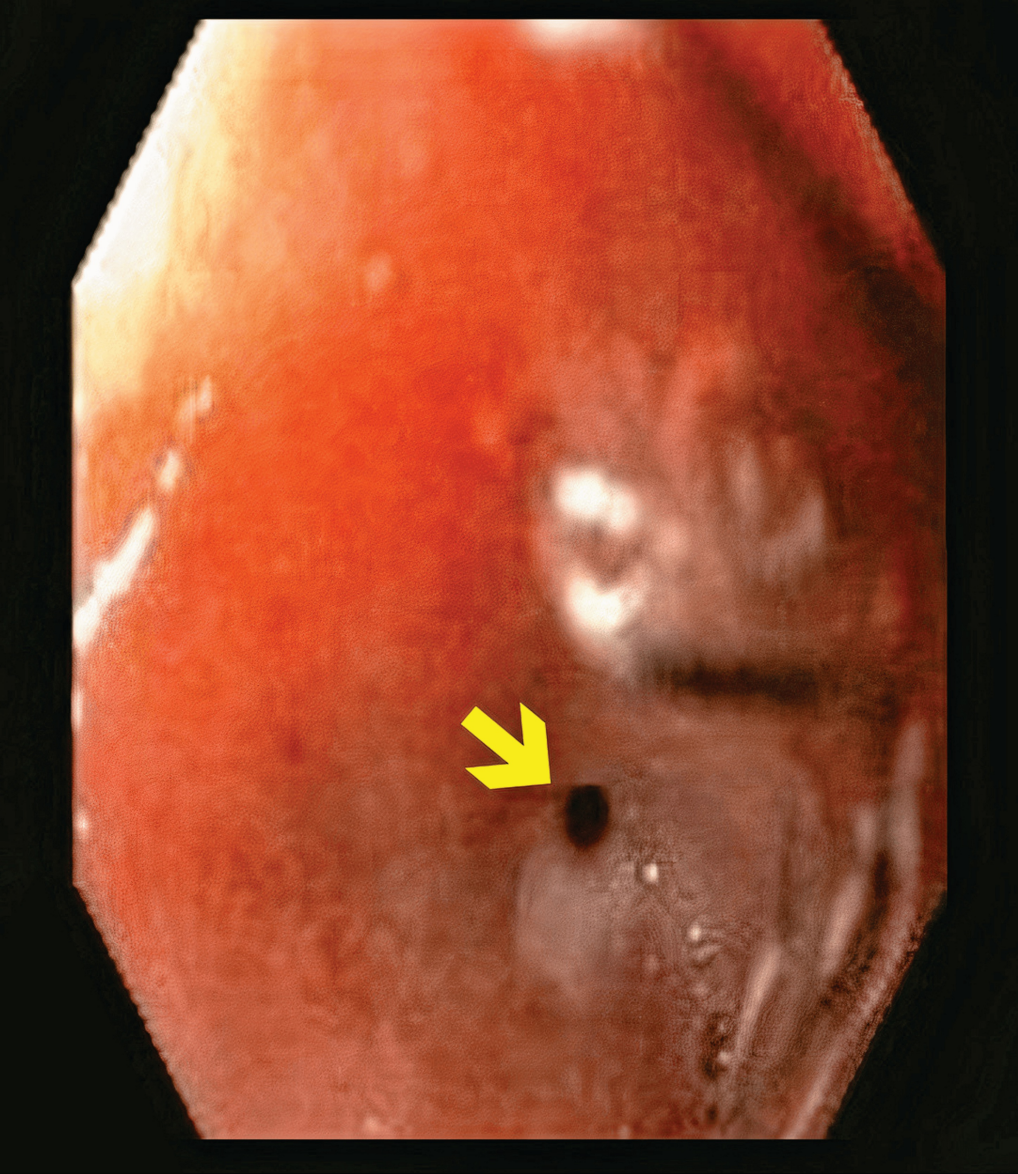
Endoscopic Evaluation After the Sixth EndoVAC Session. Marked reduction of the fistulous tract with apparent internal closure and minimal superficial opening. EndoVAC, endoscopic vacuum-assisted closure

**Figure 8:**
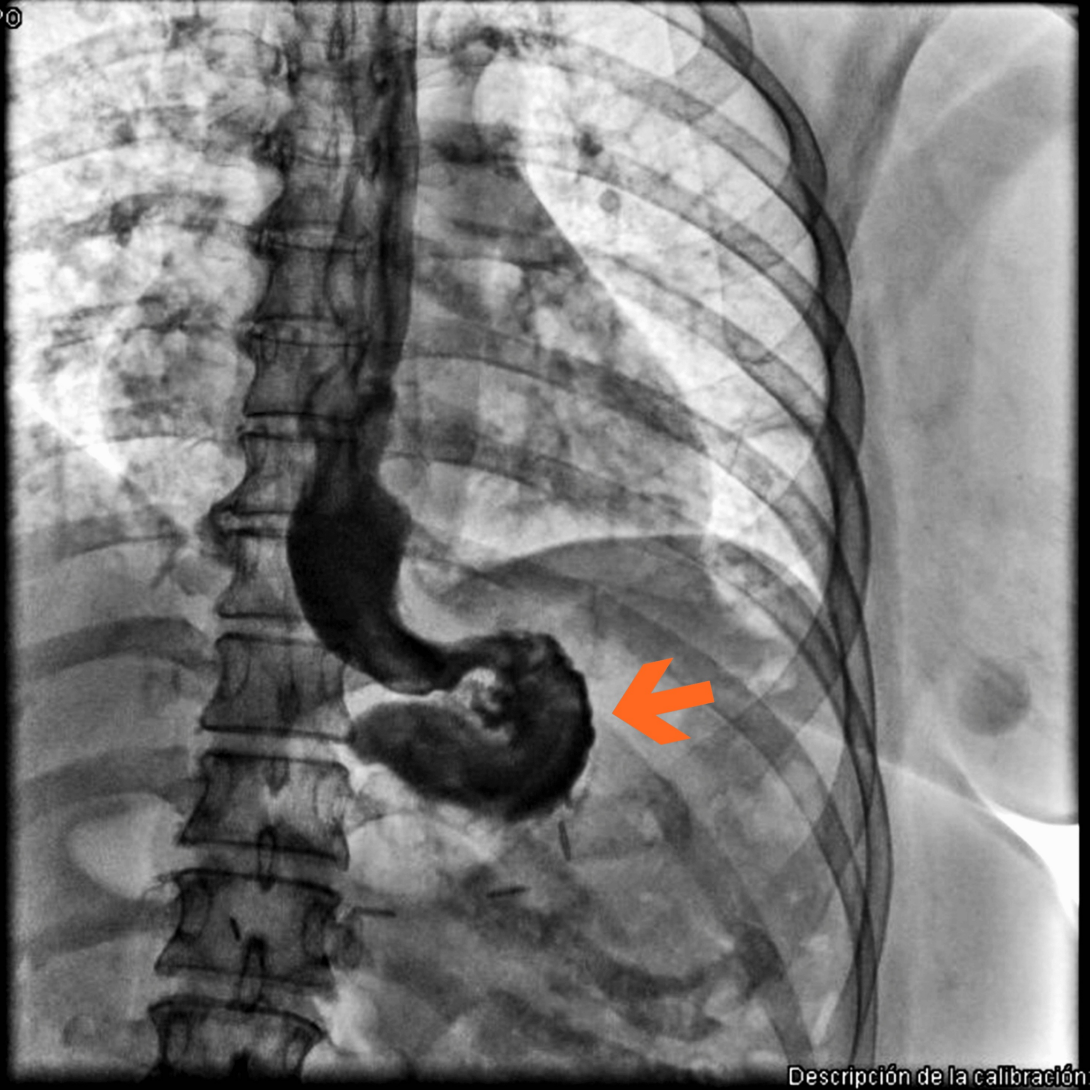
Final Contrast Study Following EndoVAC Therapy. Follow-up image using water-soluble oral contrast. No evidence of contrast extravasation through the previously identified esophagogastric tract, confirming complete closure of the fistula after six EndoVAC sessions. EndoVAC, endoscopic vacuum-assisted closure

The fistula resolved completely after six EndoVAC therapy sessions, as confirmed by follow-up endoscopy and contrast imaging (Figures 7-8). The patient was discharged in stable clinical condition and has remained asymptomatic during outpatient follow-up.

## Discussion

Sleeve gastrectomy is currently one of the most commonly performed bariatric procedures worldwide. It is associated with significant weight loss and sustained improvement in obesity-related comorbidities. However, postoperative gastric leak remains one of the most serious and concerning complications in bariatric surgery [6].

Several patient-related risk factors have been correlated with increased leak rates after sleeve gastrectomy, including male sex, BMI greater than 50 kg/m², diabetes mellitus, hypertension, obstructive sleep apnea, and smoking, as reported in recent retrospective studies [7].

In the case presented, the identifiable risk factors included a markedly elevated BMI, smoking, and obstructive sleep apnea, which are consistent with previously reported associations.

Although standard management typically includes drainage, antibiotic therapy, and more recently, endoscopic options such as over-the-scope clips (OTSCs) or endoscopic suturing, there is still no consensus regarding the most effective technique for achieving definitive closure with the lowest recurrence rates [8].

In this context, EVT (or EndoVAC) has emerged as a promising therapeutic alternative. A multicenter study on post-bariatric gastric leaks reported a 100% closure rate when EVT was used as the sole modality, with no associated mortality. The treatment duration was significantly shorter with primary EVT (17 days) compared to secondary EVT (61 days) [9]. Furthermore, a retrospective quality improvement study involving 156 patients treated with EVT for upper gastrointestinal tract leaks demonstrated significant improvement in clinical outcomes over a 10-year period. The therapeutic efficacy of EVT increased from 80% to 91%, with a marked reduction in the need for additional procedures (from 49.9% to 29.9%) and surgical reinterventions (from 38.0% to 15.6%). Moreover, the duration of leak therapy was reduced from 25 to 14 days, and the average hospital stay decreased from 38 to 25 days. Morbidity was also reduced, and a higher percentage of patients were discharged with oral nutrition. These findings support EVT as a safe and effective therapeutic strategy, allowing for faster and more functional clinical recovery in patients with esophagogastric leaks [10]. Additionally, case series using novel devices such as the VACStent have achieved 100% closure in small cohorts, without major complications [11].

In the case we report, six EVT sessions led to complete closure of the esophagogastric fistula, confirmed by endoscopy and contrast imaging, without the need for additional surgery. This outcome aligns with and reinforces the efficacy reported in previous studies, even in anatomically complex situations. The patient tolerated the treatment well and experienced no adverse events, highlighting the safety of this technique when administered by trained personnel in appropriately equipped centers.

This report also emphasizes that while EVT is promising, it is not a universal solution. Its success, in this case, does not eliminate the need for rescue surgery when endoscopic therapy fails after multiple attempts, as highlighted by Deffain et al. [12]. Furthermore, EVT requires specific resources, including the availability of sponges, continuous suction systems, and scheduled follow-up endoscopies, which may limit its implementation in healthcare settings with limited infrastructure.

Based on this case and previous data, we recommend early consideration of EVT in post-sleeve gastrectomy leaks, particularly in cases where stent placement fails or results in migration. In addition, it is essential to perform follow-up endoscopy every three to five days to reassess the defect and adjust the treatment plan accordingly. Ultimately, the development of multidisciplinary protocols in bariatric centers with access to EVT can improve patient outcomes and potentially avoid more invasive surgical interventions. 

## Conclusions

EndoVAC enabled the successful management of a postoperative esophagogastric fistula following sleeve gastrectomy in a patient with multiple risk factors and surgical complications. This approach avoided the need for surgical reintervention and achieved complete fistula closure through a gradual and clinically stable progression. The clinical experience presented in this case suggests that EndoVAC may represent an effective and minimally invasive option for treating refractory leaks unresponsive to conventional methods and highlights the importance of considering its early use as part of the treatment algorithm in selected scenarios.
